# Systematic Worldwide Review on the Performance of Non-Invasive Exhalation-Based Methods for the Diagnosis of Liver Fibrosis

**DOI:** 10.3390/ijerph23060701

**Published:** 2026-05-26

**Authors:** Jeel Moya-Salazar, Gianella S. Liviapoma-Rojas, Carlos Aliaga-Refulio, Eliane A. Goicochea-Palomino, Maria Jesús Moya-Salazar, Marx E. Morales-Martinez, Dante Espinoza-Morriberrón

**Affiliations:** 1 Faculty of Medicine, Universidad Señor de Sipan, Chiclayo 14002, Peru; a2022103487@uwiener.edu.pe; 2 School of Biomedical Engineering, Faculty of Engineering, Universidad Tecnológica del Perú, Lima 51001, Peru; 1621221@utp.edu.pe (G.S.L.-R.); 1532803@utp.edu.pe (C.A.-R.); c13136@utp.edu.pe (D.E.-M.); 3 Faculties of Health Science, Universidad Tecnológica del Perú, Lima 51001, Peru; 4 Infectious Unit, Nesh Hubbs, Lima 51001, Peru; 5 Faculty of Medicine, Universidad Nacional Mayor de San Marcos, Lima 51001, Peru; marxmoralesm@gmail.com

**Keywords:** VOC, liver fibrosis, e-nose, non-invasive, biomarker, respiratory footprint

## Abstract

Liver fibrosis is a chronic disease diagnosed through invasive methods that can worsen patients’ health. Moreover, this disease is diagnosed in terminal stages, when the damage is already widespread and irreversible, which makes it necessary to have minimally invasive diagnostic methods with high performance. The aim was to compare research on non-invasive methods, respiratory footprint, and volatile organic compounds for the diagnosis of liver fibrosis through patient exhalation. Following the PRISMA guidelines, systematic searches were conducted in 13 databases. We could identify 17,454 documents between 2009 and 2022. Inclusion criteria comprised original investigations using Gas Chromatography–Mass Spectrometry (GC-MS), Ion Mobility–Mass Spectrometer (IMR-MS), and e-nose for liver fibrosis diagnosis. We considered the precision, specificity, and sensitivity of each test and the methodological quality of each study according to the PEDro guideline. Seven investigations were included. Four (57%) studies used GC-MS, and two (28.6%) used e-nose. The most commonly used gold standard was liver biopsy, and all studies were of European origin, with only adult populations. Three (42%) studies had a specificity >90%, and five (71.4%) had a sensitivity between 85 and 100%. Isoprene is the most significant and distinguishable biomarker for liver fibrosis diagnosis. Five (71.4%) studies had high methodological quality. GC-MS is the most used technique for detecting liver fibrosis, and isoprene is the most frequent volatile organic compound (VOC) found in the exhalation of patients with liver fibrosis. More studies are needed in areas with high risk and prevalence of hepatic fibrosis.

## 1. Introduction

Liver fibrosis is a characteristic process in chronic liver diseases where scar tissue forms in the liver, which can lead to degeneration, necrosis, and hepatocellular carcinoma in advanced stages [1]. It is important to note that chronic liver diseases affect more than 1.5 billion people, and in over 50% of cases, they are detected in late stages when the damage is generally irreversible [2]. According to the World Health Organization [3], liver cancer was the third leading cause of cancer death in 2020, with approximately 830,000 deaths. Considering this and acknowledging the liver’s self-regenerative capacity, early diagnosis of fibrosis is crucial to provide timely initiation of treatment that can halt disease progression or even reverse it [2]. The gold standard method for its diagnosis is liver biopsy; however, it has risks due to being an invasive method that entails possible complications (associated with morbidity and mortality), relatively high costs, not always predicting disease progression, sampling errors, and difficulty in repeating procedures [1,4].

Given the disadvantages of liver biopsy, several non-invasive tests have been developed that can be easily and repeatedly performed on the same patient. One of the main alternatives is biological evaluation using serum biomarkers [1]. This is where human breath has emerged as a promising medium since it contains a wealth of clinically relevant biological information, with over 1000 volatile organic compounds (VOCs) carrying pathogens [2]. Exhaled breath analysis thus emerges as a promising diagnostic method for liver diseases due to its simplicity, portability, cost-effectiveness, high sensitivity, rapid response, and real-time monitoring capability [5,6].

Each of the non-invasive breath analysis techniques presents key differences in several technical and operational aspects. These include overall and maintenance costs, equipment accessibility, required professional training, analysis software, sensitivity, and sampling dimensions [6]. One of these techniques is e-nose, which uses a set of gas sensors to identify a complete profile of VOCs, albeit without specific quantification [7]. Isoprene and acetone are key VOCs that indicate disease presence. Their levels change notably in liver fibrosis, signaling disruptions in liver metabolism and increased oxidative stress. Especially, isoprene stands out as a promising non-invasive biomarker for advanced fibrosis, connecting metabolic dysfunction and oxidative stress to the progression of the disease [8,9].

Currently, two relatively new techniques are being used in research to diagnose pulmonary, gastrointestinal, and other organ diseases directly related to patient metabolism. The first is Gas Chromatography–Mass Spectrometry (GC-MS), which uses a carrier gas to separate molecules of different compounds through a column and a detector to identify volatile organic compounds [6]. The second is ion mobility-mass spectrometry/multi-capillary column (IMS/MCC), which can almost unequivocally identify substances, detect and quantify concentrations in parts per million (ppm) and parts per billion (ppb), providing structural information about the molecules analyzed [10]. This technique surpasses the capabilities of a gas chromatograph alone, as it vaporizes substances of different volatilities, ionizes neutral molecules in the gas phase, separates ions according to the charge-mass ratio, and detects and records formed ions [10].

In the context of developing non-invasive techniques for analyzing exhaled gases, the potential pros and cons of each method are evident. Therefore, it is crucial to understand their performance in diagnosing liver fibrosis to use the best method in the clinical context, achieving safe and quality care. This review aimed to compare research on non-invasive methods, respiratory footprint, and VOCs for the diagnosis of liver fibrosis through patient exhalation. Given that liver diseases remain important global health challenges, understanding and perfecting these diagnostic tools can have a significant impact on patient outcomes.

## 2. Materials and Methods

### 2.1. Study Design, Data Sources, and Search Strategy

**Figure 1:**
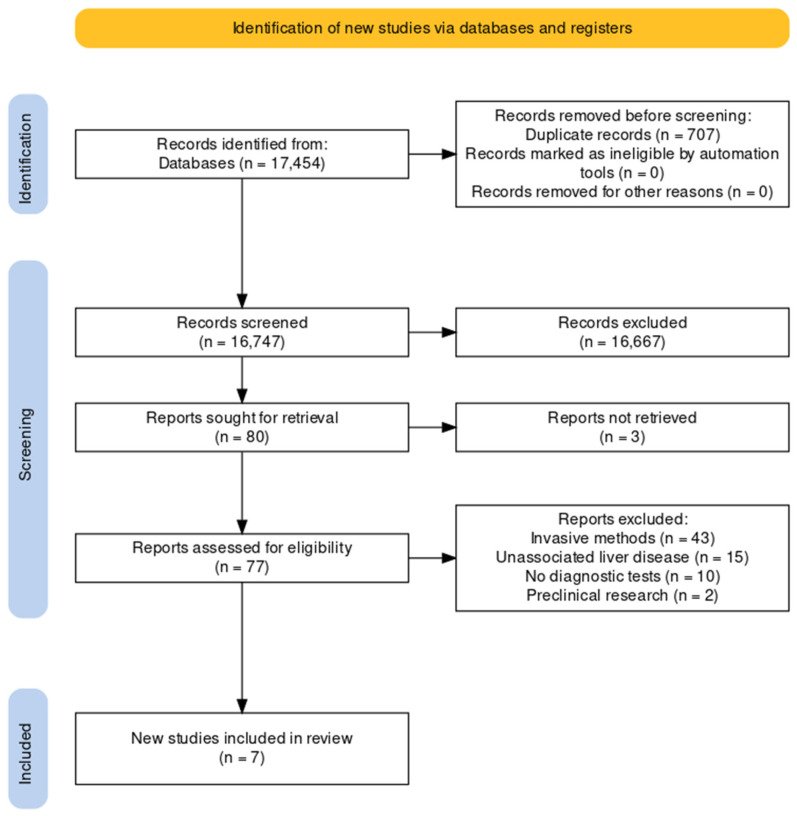
PRISMA flow diagram.

This systematic review followed the Preferred Reporting Items for Systematic Reviews and Meta-Analyses (PRISMA) 2020 guidelines [11] (Supplementary File S1) and was registered in the International Prospective Register of Systematic Reviews (PROSPERO) with the code: CRD 1117918. Searches were conducted in seven databases (PubMed/Medline, Scopus, ScienceDirect, Scielo, Latindex, LILIACS, and EBSCO), two preprint servers (medRxiv and Preprints), three thesis repositories (Alicia CONCyTec, RENATI, and Colciencias), and Google Scholar between 10 and 15 December 2022 (Figure 1). The search was conducted using the following equation “((Exhaled breath analysis OR volatile organic compounds OR VOC) AND (hepatology) NOT (COVID-19 OR SARS-CoV-2))” with no language restrictions and adapted to the specific requirements of each database (Supplementary File S2).

### 2.2. Inclusion and Exclusion Criteria

The inclusion criteria were (i) original research, cohort studies, case–control studies, clinical trials, short communications, commentaries, and theses; (ii) participants of both genders over 18 years old; (iii) research using GC-MS (Gas Chromatography–Mass Spectrometry), IMR-MS (Ion Mobility–Mass Spectrometer), and e-nose for the diagnosis of hepatic fibrosis; (iv) research with diagnostic performance analysis, and (v) published between 2009 and 2022. Studies on COVID-19 and fibrosis or liver diseases were excluded, as well as systematic reviews, editorial letters, historical and reflection articles, case reports, erratum, methodological papers, research without a control group or without liver disease.

### 2.3. Selection and Document Extraction

Following the PRISMA protocol, all processes were manual. Thus, abstracts were independently evaluated by three authors (G.S.L.-R., C.A.-R., and J.M.-S.) and excluded if they did not meet the inclusion criteria, with studies that were reviewed in full text being included after consensus meetings where disagreements between reviewers were reviewed and resolved [12]. The Kappa correlation test was used to determine overall agreement between reviewers (κ = 0.899) [13]. Research directly related to breath analysis, hepatology, hepatic fibrosis, and VOCs in breath were considered.

### 2.4. Assessment of Research Quality

We used the PEDro (Physiotherapy Evidence Database) guide to measure the methodological quality of experimental studies [14]. These tools have been widely used in reviews and have provided an overview of the quality of selected research [15,16]. To assess quality, the recommended protocol was considered [17], which considers high-quality methodology with a score of at least 7 and medium quality with a score of 5 and 6. Disagreements in methodological quality analysis were resolved by consensus among the authors in three weekly meetings.

### 2.5. Data Extraction and Analysis

Data extraction was manual and initially performed on a spreadsheet in MS-Excel 2013 (Microsoft Corp., Redmond, WA, USA). We used IBM SPSS version 23.0 (Armonk, NY, USA) for descriptive analysis (mean, SD) of the included studies, estimating simple frequencies and measures of central tendency. Additionally, data were extracted and reported directly on diagnostic performance, including sensitivity, specificity, positive predictive value (PPV), negative predictive value (NPV), Positive and Negative Likelihood (LR+/LR−), and AUROC (Area Under the Receiver Operating Characteristic). Furthermore, the frequency of VOC types in parts per billion (ppb) was analyzed. Since the works were heterogeneous regarding the type of sample analyzed against the control test, a meta-analysis could not be performed (i.e., heterogeneity in methods and lack of complete results), and a narrative synthesis of the research is presented.

## 3. Results

The initial search yielded 17,454 documents, of which 490 were preprints and 34 theses. Seventy duplicate documents were removed, and 16,670 were excluded for not being related to the objective and topic of the review (liver fibrosis diagnosis). Thus, 77 documents were reviewed in full text, and 69 were excluded for not meeting the selection criteria (i.e., 43 documents of invasive methods). Finally, 7 documents were included in the review [7,8,18,19,20,21,22] (Figure 1).

### 3.1. Study Characteristics

Of the total included documents, three were identified in PubMed/Medline [7,19,21], three in Google Scholar [8,20,22], and one from ScienceDirect [18]. According to the countries where they were conducted, two studies were from the UK [18,19], two from Germany [21,22], one from Italy [7], one from Belgium [20], and one from the United States [8] (Table 1). Only one study was published in 2008 [20], from 2010 to 2020, we found five published studies [7,8,18,19,22] and from 2021 to 2022, there is only one published research [21]. In total, 624 individuals were included in this systematic review. De Vincentis et al. [7] presented the largest sample size with 160 individuals, and Voss et al. [21] presented the smallest number of participants with 30 individuals. All studies considered only adult participants (34–80 years) and were exploratory.

### 3.2. Comparison of Non-Invasive Methods for Liver Fibrosis

#### 3.2.1. Methods and Gold Standard Tests for Liver Fibrosis

Four out of seven studies (57%) used GC-MS [16,18,19,20], two used e-nose [7,18], and only one used IMR-MS [22]. As a diagnostic gold standard test, three out of the seven studies (42%) used hepatic biopsy [8,19,20], two (28%) used ultrasound [7,22], and only one used serum liver function analysis [21]. All were used as primary gold standard tests. Three studies did not present a secondary gold standard test [8,18,21], two used ultrasound [7,19], and one [22] used clinical history and blood liver function analysis. Finally, Van del Velde et al. [20] used biochemical and radiological tests that were not detailed, which limited comparability.

#### 3.2.2. Performance of Non-Invasive Methods for Liver Fibrosis

Three out of seven studies (42%) had a specificity of less than 90% [8,18,20]. Four studies had a specificity in the range of 90–100% [7,19,21,22]. Two (28.6%) out of the seven studies had a sensitivity of 100% [20,21], and the other 5 (71.4%) studies had a sensitivity in the range of 85–100% [7,8,18,19,22] (Average sensitivity: 93.02%). Five out of the seven studies (71%) had an Area Under the Receiver Operating Characteristic Curve (AUROC) value in the range of 80–100% [7,8,18,21,22] (Table 2). In the studies included for non-invasive detection of liver fibrosis, the sensitivity of tests based on GC-MS [8,19,20,21] was higher than 85%, while e-nose had 86.2% and 88% as lower sensitivity results in the studies by De Vincentis [7] and Arasaradnam [18], respectively.

#### 3.2.3. VOC’s Frequency

Only three out of the seven studies (42%) [8,20,22] have quantifiable data on VOCs. Isoprene is a compound present in two studies [8,22], and in both studies (Table 3), an increase in this compound is evident in the participants of the test group compared to the control group. Acetone is another compound present in two studies [8,20] with a notable difference in the amount of VOC between the control and test groups.

In the study by Alkohouri et al. [8], four VOCs were detected (Isoprene, Acetone, Pentane, Ethanol). Between the test group and the control group, Isoprene showed an increase of 33%; additionally, another VOC with a significant increase was Acetone (approximately 52% compared to the control group). In the study by Millonig et al. [22], three VOCs were detected (Acetaldehyde, Ethanol, and Isoprene). Isoprene showed an increase of 37% compared to the control group. Lastly, in the study by Van del Velde et al. [20], more complex VOCs were found compared to the other two studies described previously, but the most differentiated VOC was Acetone with an increase of almost 65%.

#### 3.2.4. Quality Assessment and Bias

The methodological quality of the studies ranged between values 6 and 7. Five out of (71%) studies [7,8,18,19,20] had high methodological quality, and the other two remaining studies [21,22] had medium quality. Additionally, according to PEDro, the methodological quality of all studies was medium to high. While the analyzed studies appeared to be homogeneous, there were some criteria that were not consistent with the rest. All research [7,8,18,19,20,21,22] met criteria 1, 7, 8, 9, and 10; however, three criteria—3, 5, and 6—were not met by any of the seven studies (Table 4).

## 4. Discussion

This systematic review, conducted in 624 adult participants, found that most studies use GC-MS for non-invasive detection of liver fibrosis with a sensitivity and specificity exceeding 80%. These studies were conducted in the last 10 years, have an exploratory design, were mainly carried out in European countries, and have a medium-to-high methodological quality and bias assessment according PEDro tool.

### 4.1. Strengths

This systematic review is one of the first to include non-invasive, exhaled air-based methods for detecting liver fibrosis, such as VOC analysis, and to evaluate each method’s performance against a reference standard. However, due to the variability in the available evidence, the reference standards used were inconsistent, which may affect the comparability of the results. There are systematic reviews and research on liver fibrosis that have described risk factors [23], quantified the costs and effectiveness of early treatment [24], evaluated the performance of histopathological methods versus imaging [25], and described new updates on its classification and staging [26]. However, they have not compared non-invasive diagnostic methods. Another strength of the study is the number of scientific search engines used, including meta-search engines, thesis repositories, and preprint servers. The inclusion of these bibliographic sources substantially improves the comprehensiveness of the review [27] and permits us to find research hosted in uncommon search engines considered gray literature. It is noteworthy that this study is a significant contribution to researchers seeking to improve the technology for detecting VOCs during exhaled breath testing in hepatic fibrosis [28].

### 4.2. Main Findings

In the seven studies we analyzed, there was heterogeneity in the Gold Standard test for detecting liver fibrosis with non-invasive methods. The most used test was biopsy [8,19,20]; however, due to its invasiveness and the risk of morbidity and mortality it has, it is becoming less used. Ultrasound was the second most used test [7,22], and lastly, liver function analysis, which encompasses a series of analyses of both blood and other visible symptoms [21]. The sensitivity of the studies included for detecting liver fibrosis through non-invasive methods by exhaled breath testing was greater than 85%, which is comparable to another study from Belgium [29] that had a sensitivity of 85% for detecting malignant pleural mesothelioma with GC-MS. The high sensitivity found in studies to detect liver fibrosis and pleural mesothelioma is subject to the sample quality and the analyzed biomarkers (e.g., time of collection, patient fasting and absence of cigarette smoking, rest before the measurements, etc.) [18,20]. On the other hand, the specificity of studies with GC-MS was in the range of 68–100%, similar to another study from Belgium [30] that had a specificity of 97%. The specificity of the previously described studies maintains a high value due to the quality of the methodological design used and the quantitative analysis of VOCs.

According to our findings, four studies used GC-MS to diagnose or differentiate the severity or progression of fibrosis in patients with chronic liver disease. The most recent study, conducted by Voss et al. [21], used Metal Oxide (MOx) gas sensors for the differential diagnosis of the severity of liver dysfunction, achieving a sensitivity and specificity of 100% to distinguish advanced states of fibrosis, such as compensated and decompensated cirrhosis, from healthy patients. This level of accuracy was attained by assessing just one exhalation cycle from the patient, offering an advantage in the diagnostic process. A similar approach was seen in Khalid et al.’s study [19], which used multivariate discriminant analysis to identify VOCs and differentiate patients according to disease status, reporting a sensitivity of 92.3% and 97.1% for alcoholic and nonalcoholic cirrhosis from healthy controls, respectively. However, neither of these two studies described the identified VOCs.

Van del Velde et al. [20], on the other hand, investigated specific compounds in patients with hepatic cirrhosis, detecting 12 compounds, among which acetone, 2-butanone, 2-pentanone, and dimethyl sulfide were significantly higher in the breath of these patients. Similarly, Alkohouri et al. [8] used VOC levels as biomarkers to specifically detect advanced fibrosis. These patients showed significantly lower values in six compounds compared to the rest (*p* < 0.002), with isoprene being the most accurate for prediction (Area Under the Receiver Operating Characteristic (AUROC): 0.855, 95%CI: 0.762–0.948), with a mean level of 13.5 ppb in patients with advanced fibrosis versus 40.4 ppb in patients with fibrosis (*p* < 0.001). This finding was considered by Mehaney et al. [28], who proposed the use of photonic crystals in sensor structures to improve the detection of isoprene concentration in exhaled breath. This theoretical and computational design did not involve patient testing, and its results showed high sensitivity. Therefore, it highlights the progress of technology in non-invasive methods as a way to reduce the risk of infection during blood sampling with a syringe for hepatic fibrosis detection.

The e-nose allows real-time differentiation and distinction between patients with specific diseases and healthy individuals; however, it does not specifically quantify the detected chemicals. Despite this, Arasaradnam et al. [18] and De Vincentis et al. [7] have highlighted this technology as a means for early diagnosis of hepatic fibrosis, which could prevent progression to more severe stages of the disease, where damage is often irreversible [2]. Our results have shown a sensitivity exceeding 80% for the e-nose, demonstrating significant performance that could also be used to monitor the patient’s response to various treatments [7].

Only Millonig et al. [22] utilized IMR-MS combined with a novel statistical approach aiming (Stacked Feature Ranking (SFR)) to enhance diagnostic accuracy during exhaled breath analysis in liver diseases. Their study demonstrated that acetaldehyde was comparable between healthy individuals and those with hepatic cirrhosis, while significantly elevated in patients with alcoholic or non-alcoholic fatty liver disease. Ethanol only increased in patients with hepatic cirrhosis, and isoprene significantly increased in those with alcoholic fatty liver disease compared to all other groups, thus highlighting the method’s specificity in identifying fibrosis stages as the disease progresses. The authors also noted two potential interferences in this procedure. Firstly, the patient’s diet may influence breath composition; hence, it is necessary to conduct the test after an overnight fast for more reliable data. Secondly, smoking is also an important factor, as some compounds can be detected up to a week after the last cigarette. Therefore, Millonig et al. [22] selected similar percentages of these compounds present in smokers and applied them across their entire sample, thus eliminating the influence of these molecules on exhaled breath results.

This highlights once again how advancements in the specificity of these non-invasive methods, through the measurement of biomarkers in exhaled breath, can improve the diagnosis of liver diseases without tests being confounded by patient lifestyle factors such as diet and smoking. Furthermore, they allow early diagnosis for timely intervention to halt or reverse the disease process, not only through pharmacological treatments or therapy but also by modifying lifestyles (e.g., reduced alcohol intake, healthier diet), thereby reducing the burden of healthcare [2].

All our evidence on noninvasive methods using VOCs and respiratory fingerprinting to diagnose liver fibrosis through exhalation is from Europe and the United States, with little data from Asia, Africa, and Latin America. For instance, studies that identify distinct VOC profiles and create classification models to distinguish cirrhosis patients from healthy controls mainly originate from European and American institutions [31,32]. Other methodological and technical reviews have also highlighted the lack of standardization and the need for multicenter and multinational studies to validate these methods in different epidemiological and ethnic contexts [33,34]. Multicenter and global studies are required to validate these technologies and adapt them to diverse populations, as the prevalence, severity, progression, and access to healthcare in these regions greatly influence the potential performance of liver fibrosis diagnostic technologies.

Our data show that exhalation-based methods, particularly VOC analysis, show good potential for non-invasive identification of liver fibrosis [35,36,37]. However, their diagnostic performance and reproducibility need to be further validated in larger, multicenter studies. Unlike elastography, which requires expensive ultrasound or MRI equipment and trained personnel, breath analysis can be performed using portable, potentially low-cost equipment, increasing accessibility in primary care or resource-limited settings [38]. In this sense, exhalation-based methods complement elastography by offering a painless, accessible, and potentially scalable approach for liver fibrosis screening and monitoring, especially where imaging is impractical or imaging access is limited [38]. However, elastography remains the gold standard among non-invasive tools due to its robust validation and direct measurement of fibrosis. Elastography (by ultrasound or MRI-based) has been extensively validated, has high diagnostic accuracy for severe fibrosis and cirrhosis, and established clinical cutoff values [39,40]. Non-invasive tools require rigorous validation and extensive analysis before they can directly measure fibrosis.

### 4.3. Limitations

The methodological quality found in the studies is high. However, these studies are exploratory, and clinical trials need to be conducted to have a broader, more organized, and real understanding of VOC detection in the exhalation of patients with liver fibrosis [7,8,18,19,20,21,22]. Additionally, some studies [8,18,21] have a population of fewer than 100 participants, so it is necessary to improve research with a larger number of participants and conduct a meta-analytical analysis to correctly assess the performance of non-invasive technologies.

The variability among the included studies, such as using different gold standards and exploratory designs, represents a significant limitation. For better meta-analytical synthesis, future liver fibrosis research should use consistent reference standards, validated test cut-offs, standardized methodologies, and thorough reporting [36,41,42,43]. These measures will help reduce heterogeneity and enhance the reliability of systematic reviews and meta-analyses.

Although the technologies examined in this systematic review show high accuracy in detecting liver fibrosis, recently, in the United States, Owlstone Medical, a company, has developed a portable device for exhaled gas analysis called ReCIVA [31]. However, the absence of data from centers outside Europe and the US indicates that such data are not widely accessible and may have limited relevance in other settings. Therefore, more comparative international studies between these technologies are needed to determine their respective accuracy in the addressed context.

## 5. Conclusions

Most studies (4/7) use GC-MS for non-invasive detection of liver fibrosis with sensitivity and specificity exceeding 80%. The methodological quality of the studies is medium to high according PEDro assessment tool, and the most distinguished VOCs in the exhalation of patients were isoprene and acetone. These non-invasive methods (i.e., GC-MS), regardless of whether they quantify VOCs or not, enable the detection of hepatic fibrosis and its severity quickly, accurately, cost-effectively, and portably, allowing early diagnosis and intervention to improve patient monitoring, reducing the need for invasive pathological diagnosis and healthcare burden for this disease. The evidence of this study is based on small, exploratory, heterogeneous studies. The evidence of this study is based on small, exploratory, heterogeneous studies. There is an urgent need for large-scale, multicenter clinical trials to make the conclusion more realistic and forward-looking.

## Figures and Tables

**Table 1:** Baseline characteristics of the included documents.

Author	Year	Country	Population	N	Design	Phase
Alkhouri	2015 [8]	USA	Adults	61	Exploratory	Pilot
De Vincentis	2016 [7]	Italy	Adults	160	Exploratory	Proof-of-concept
Voss	2022 [21]	Germany	Adults	30	Exploratory	Pilot
Arasaradnam	2016 [18]	UK	Adults	42	Exploratory	Pilot
Millonig	2010 [22]	Germany	Adults	126	Exploratory	Pilot
Khalid	2013 [19]	UK	Adults	103	Exploratory	Pilot
Van den Velde	2008 [20]	Belgium	Adults	102	Exploratory	Proof-of-concept

**Table 2:** Diagnostic performance of non-invasive methods based on exhalation.

Author	Technology Used	Study Type	Population	Diagnostic Pattern	Second Analysis Test	Performance
Alkhouri [8]	GC-MS	Exploratory	61	Biopsy	N/D	Specificity: 68% Sensitivity: 85% Positivity: 57% NPV: 90% AUROC: 86% (CI 95%: 76.2 to 95.8)
De Vincentis [7]	e-nose	Exploratory	160	Ecography	Ultrasonography	Specificity: 98.2% Sensitivity: 86.2% LR+: 48.3 LR−: 0.1 NPV: 85.9% PPV: 98.3% AUROC: 84% (CI 95%: 76 to 91)
Voss [21]	GC-MS	Exploratory	30	Liver function analysis	N/D	Specificity: 100% Sensitivity: 100% Precision: 100% AUROC: 100%
Arasaradnam [18]	e-nose	Exploratory	42	Liver function analysis	N/D	Specificity: 68% (CI 95%: 51 to 81) Sensitivity: 88% (CI 95%: 73 to 95) AUROC: 84% (CI 95%: 75 to 93)
Millonig [22]	IMR-MS	Exploratory	126	Ecography	Medical history and blood analysis	Specificity: 100% Sensitivity: 95% AUROC: 97% (CI 95%: 92 to 100)
Khalid [19]	GC-MS	Exploratory	103	Biopsy	Ultrasonography	Specificity: 93% Sensitivity: 97% LR+:14.56 LR−: 0.03 NPV: 93% PPV: 97%
Van den Velde [20]	GC-MS	Exploratory	102	Biopsy	Biochemical and radiological	Specificity: 70% Sensitivity: 100%

Abbreviation: GC-MS: Gas Chromatography–Mass Spectrometry, IMR-MS: Ion Mobility–Mass Spectrometer, AUROC: Area Under the Receiver Operating Characteristic, LR+: Positive Likelihood Ratio, LR−: Negative Likelihood Ratio, NPV: Negative Predictive Value, PPV: Positive Predictive Value.

**Table 3:** Diagnostic analysis of VOC in non-invasive exhalation-based methods.

Author	Year	Country	Population	N	Type	VOCs Frequency		
						VOCs	Control (ppb)	Test (ppb)
Alkhouri	2015 [8]	USA	Adults	61	Exploratory	Isoprene: Acetone: Pentane: Ethanol:	13.5 117.8 12.3 63	40.4 224.2 19.5 75.6
Millonig	2010 [22]	Germany	Adults	126	Exploratory	Acetaldehyde: Ethanol: Isoprene:	180 900 45	350 1000 120
Van del Velde	2008 [20]	Belgium	Adults	102	Exploratory	Acetone: 2-pentanone: 2-butanone: Dimethyl:	212.25 0.38 0.38 13.79	325.92 0.61 1.42 23.24

Abbreviation: VOC: Volatile Organic Compound, ppb: parts per billion.

**Table 4:** Methodological quality according to PEDro of the included documents (*n* = 7).

Study	1	2	3	4	5	6	7	8	9	10	11	Score	Study Quality
Alkhouri et al. (2015) [8]	+	−	−	+	−	−	+	+	+	+	+	7	High
De Vincentis et al. (2016) [7]	+	−	−	+	−	−	+	+	+	+	+	7	High
Voss et al. (2022) [21]	+	+	−	+	−	−	−	+	+	+	?	6	Medium
Arasaradnam et al. (2016) [18]	+	−	−	+	−	?	+	+	+	+	+	7	High
Millonig et al. (2010) [22]	+	−	−	−	−	−	+	+	+	+	+	6	Medium
Khalid et al. (2013) [19]	+	−	−	+	−	−	+	+	+	+	+	7	High
Van den Velde et al. (2008) [20]	+	+	−	+	?	−	+	+	+	+	−	7	Medium

The column numbers correspond to the following descriptions of the PEDro scale: 1. Eligibility criteria were specified. 2. Subjects were randomly allocated to groups. 3. Allocation to groups was concealed. 4. Groups were similar at baseline regarding the most important prognostic indicators. 5. There was blinding of all participants. 6. There was blinding of all therapists who administered the intervention. 7. There was blinding of all assessors who measured at least one key outcome. 8. Key outcome measures were obtained from more than 85% of subjects initially allocated to groups. 9. All subjects for whom outcome measures were available received the treatment or control condition as allocated, or, if this was not the case, data for at least one key outcome was analyzed on an intention-to-treat basis. 10. The results of between-group statistical comparisons were reported for at least one key outcome. 11. The point estimate and variability measures for at least one key outcome were provided by the statistician. The final score was determined by summing the items that met the established criteria, with the exception that item number 1 was not considered. “+” indicates that the item was clearly satisfied, “−” indicates that the item was not satisfied, and “?” indicates that it is unclear whether the item was satisfied or not; in any case, it does not count toward the total score.

## Data Availability

The original contributions presented in the study are included in the article/Supplementary Materials, further inquiries can be directed to the corresponding author.

## Supplementary Materials

The following supporting information can be downloaded at: https://www.mdpi.com/article/10.3390/ijerph23060701/s1, Supplementary File S1: PRISMA 2020 Main Checklist; Supplementary File S2: Search equations according to each scientific search engine. Reference [44] is cited in the Supplementary Materials.
