# Supplementary material for: Systematic Worldwide Review on the Performance of Non-Invasive Exhalation-Based Methods for the Diagnosis of Liver Fibrosis
Source: Int J Environ Res Public Health. 2026 May 26;23(6):701. doi: 10.3390/ijerph23060701 (PMC13300345; doi:10.3390/ijerph23060701)
Supplement: Supplementary file 1 [file ijerph-23-00701-s001.zip › Suppl.2_Search equations according to each scientific search engine..pdf]

Suppl. 2. Search equations according to each scientific search engine.

Search engine	Keywords	Results
PubMed 2009 - 2022	exhaled breath analysis AND hepatology	136
PubMed 2009 - 2022	exhaled breath analysis OR volatile organic compounds OR VOC OR biomarkers AND hepatology	15525
PubMed 2009 - 2022	(Exhaled breath analysis OR volatile organic compounds OR VOC) AND (hepatology) NOT (COVID-19 OR SARS-CoV-2)	177
ScienceDirect 2009 - 2022	exhaled breath analysis OR volatile organic compounds OR VOC OR biomarkers AND hepatology	610
ScienceDirect 2009 - 2022	(Exhaled breath analysis OR volatile organic compounds OR VOC) AND (hepatology) NOT (COVID-19 OR SARS-CoV-2)	5
Scopus 2009 - 2022	(Exhaled breath analysis OR volatile organic compounds OR VOC) AND (hepatology)	3
Scopus 2009 - 2022	exhaled breath analysis AND hepatology	4
Scielo	Exhaled breath analysis OR volatile organic compounds OR VOC	0
Scielo	Exhaled breath analysis	6
LILIACS	(Exhaled breath analysis OR volatile organic compounds OR VOC) AND (hepatology)	90

LILIACS	compuestos orgánicos volátiles AND análisis AND exhalado	0
Preprints	exhaled breath analysis OR volatile organic compounds OR VOC OR biomarkers AND hepatology	222
Preprints	exhaled breath analysis OR biomarkers	299
ALICIA	(compuestos orgánicos) OR (compuestos fenólicos)) volátiles Y Todos los Campos:(biomarcador OR biomarcadores))	0
ALICIA	exhaled breath analysis	3
Google Académico	(Exhaled breath analysis OR volatile organic compounds OR VOC) AND (hepatology)	374
